# Design of Chitosan and Its Water Soluble Derivatives-Based Drug Carriers with Polyelectrolyte Complexes

**DOI:** 10.3390/md12126236

**Published:** 2014-12-19

**Authors:** Qing-Xi Wu, Dong-Qiang Lin, Shan-Jing Yao

**Affiliations:** 1 Integrated Biotechnology Laboratory, School of Life Science, Anhui University, Hefei 230601, China; E-Mail: wuqx@ahu.edu.cn; 2 Key Laboratory of Biomass Chemical Engineering of Ministry of Education, College of Chemical and Biological Engineering, Zhejiang University, Hangzhou 310027, China; E-Mail: lindq@zju.edu.cn; 3 Collaborative Innovation Center of Chemical Science and Engineering (Tianjin), Tianjin 300072, China

**Keywords:** chitosan, water soluble chitosan, drug carriers, polyelectrolyte complexes, targeted/controlled release

## Abstract

Chitosan, the cationic polysaccharide derived from the natural polysaccharide chitin, has been studied as a biomaterial for more than two decades. As a polycationic polymer with favorable properties, it has been widely used to form polyelectrolyte complexes with polyanions for various applications in drug delivery fields. In recent years, a growing number of studies have been focused on the preparation of polyelectrolyte complexes based on chitosan and its water soluble derivatives. They have been considered well-suited as biomaterials for a number of vital drug carriers with targeted/controlled release profiles, e.g., films, capsules, microcapsules. In this work, an overview highlights not only the favorable properties of chitosan and its water soluble derivatives but also the good performance of the polyelectrolyte complexes produced based on chitosan. Their various types of applications as drug carriers are reviewed in detail.

## 1. Introduction

With nonrenewable resources running out all over the world, more and more polysaccharides from the natural world have been explored as advanced functional biomaterials and new energy resources, especially in recent years. Among them, chitin is known as the second most abundant renewable polymer in nature next only to cellulose. Chitin extensively exists in the exoskeleton of crustaceans, e.g., crab shells, lobsters, shrimp. It can also be found in mollusk radulas, cephalopod beaks, insects, fungal cell walls. Chitosan is considered a cationic polysaccharide, which is obtained from chitin following an alkaline deacetylation. With remarkable structural and functional properties, chitosan and its water soluble derivatives have been concerned by researchers in fundamental science and industry application.

**Figure 1:**
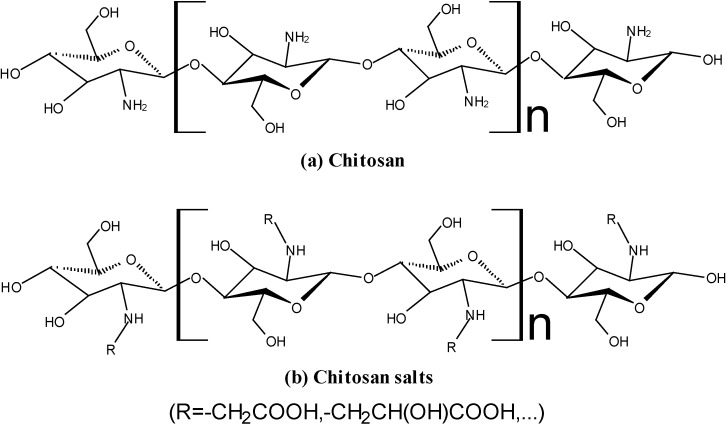
Structure of chitosan and chitosan salts.

Chitosan is a linear copolymer composed by glucosamine and *N*-acteyl glucosamine units, via β-(1,4) linkages, namely 2-amino-2-deoxy-β-d-glucan (GlcN) (Figure 1a). It is the product of the deacetylation reaction of chitin (2-acetamido-2-deoxy-β-d-glucan (GlcNAc)). Chitosan is able to dissolve in acidic solutions, becoming a polycationic polymer with a high density of positive charges (–NH_3_^+^ groups). It has favorable biological properties, such as no-toxicity, mucoadhesiveness, biocompatibility and, more importantly, the biodegradability, which means it could be digested by the bacteria in the human colon [1,2,3]. Recently, the water soluble derivatives of chitosan, hereafter water soluble chitosan (WSC), such as chitosan salts (Figure 1b), zwitterionic chitosan and chitosan oligomers, have drawn increasing attention due to their water-solubility [4,5,6,7,8,9,10]. WSC has the similar favorable properties with chitosan but can be dissolved in neutral aqueous media. Therefore, both of them are challenging biomaterials with potential vital applications in bioengineering and biopharmaceutical fields [11,12,13,14].

As a macromolecule with positive charges, chitosan and WSC can chemically conjugate with a lot of anionic substrates forming polyelectrolyte complexes (PEC). These anionic substrates include both polyanionic polymers and small molecular substances, such as sodium alginate [15,16], hyaluronic acid [17], sodium cellulose sulfate (NaCS) [18], glutaraldehyde [19], genipin [20]. Based on chitosan and WSC, the materials normally used for the preparation of PEC to design new drug delivery carriers can be seen in Table 1.

**Table 1:** Materials normally used for the preparation of polyelectrolyte complexes (PEC) based on chitosan and water soluble chitosan (WSC).

Polycationic Polyelectrolyte	Polyanionic Polyelectrolyte	Cross-Linking Agent	Preparation Method	Package Drugs	Reference
Chitosan	Sodium alginate	Calcium chloride	Coacervation	Rifampicin	[15]
Chitosan	Hyaluronic acid	TPP	Ionotropic gelation	Heparin	[17]
Chitosan	NaCS	—	Dipping-process	5-ASA	[18]
Chitosan	Carrageenan	Glutaraldehyde	Complex coacervation	*Pimenta-dioica* oil	[19]
Chitosan	Carboxymethyl cellulose	Genipin	*In situ* synthesis	—	[20]
Chitosan	Pectin	—	Wet granulation	Theo-phylline	[21]
Chitosan	Xanthan gum	—	Hot-melt extrusion	CPM	[22]
Chitosan	—	Polyethylene glycol	Emulsification	5-FU	[23]
WSC^a^	Poly-(l-aspartic acid)	—	Coagulation	BSA	[9]
WSC^b^	NaCS	PPS	Orifice-polymerization	Lactoferrin	[24]

TPP: pentasodium tripolyphosphate; NaCS: sodium cellulose sulfate; 5-ASA: 5-aminosalicylic acid; CPM: chlorpheniramine maleate; 5-FU: 5-fluorouracil; WSC^a^: chitosan with molecular weight 6 kDa and deacetylation degree 0.93; BSA: bovine serum albumin; WSC^b^: chitosan hydrochloride; PPS: Sodium polyphosphate.

The prepared PEC based on these substances exhibit favorable biological performances, such as a definite hydrophilic and swellable character, low interfacial tension and high permeability, favorable film-forming behavior, excellent biodegradability and good biocompatibility [25]. Based on these favorable performances, PECs have received the attention of more and more researchers for the preparation of drug carriers or tissue engineering scaffolds [26,27,28,29]. The details will be presented in the next sections of this review.

## 2. Properties of Chitosan and WSC

### 2.1. No-Toxicity

Chitosan is a well-known approved pharmaceutical excipient with no or low toxicity [30]. Chitosan has also been approved by the US Food and Drug Administration (FDA) for use in wound dressings [31] and is used as dietary additives in Japan, Italy and Finland [32]. Despite the lethal dose presented by chitosan as LD_50_ = 16 g/kg body weight when orally administered to mice, this level has been shown to be biodegradable [30,33]. In another study, Costa *et al*., showed that chitosan-based mouthwash possessed no genotoxicity and lower cytotoxicity than the commercial mouthwash [34]. Meanwhile, alcohol-free mouthwash based on water-soluble chitosan has also proved to have no cytotoxicity [35]. It had also been confirmed that the toxicological side effects of chitosan are dependent on the molecular weight, degree of deacetylation and charge density of the molecule, specifically the toxicity is related to the molecular weight when at a high degree of deacetylation and it increases with increasing density [36].

### 2.2. Solubility

Chitosan is insoluble at neutral and high pH regions due to its molecular structure and p*K_a_* (6.2–7.0) [37,38]. It means that chitosan can be protonated at low pH in aqueous solutions [39]. Therefore, acidic solvents, such as diluted solutions of acetic acid (1%–3%, v/v) and citric acid (3%–4%, v/v) are usually needed to prepare chitosan solutions. However, the derivatives of chitosan, WSC can be directly dissolved in water under neutral pH conditions. It makes the process simpler while avoiding the use of acidic solvents; therefore WSC had received the attention of more and more researchers [37,40,41,42,43,44].

### 2.3. Biocompatibility

Chitosan has been widely used in the biomedical field, as it has already proved to be highly biocompatible [45,46]. Additionally, as a pharmaceutical excipient, WSCs like chitosan hydrochloride were approved by the European Pharmacopoeia (4th edition, 2002). Further, Marsiyana *et al*., verified that the chitosan-bound microtubes were highly biocompatible and the experimental cells were able to survive and proliferate at a similar rate as the control [47]. Besides, the chitosan derivative named zwitterionic chitosan (ZWC), which is soluble in water at pH’s below and above the p*I*, showed an excellent compatibility with the blood components and a good toleration upon an intraperitoneal (IP) injection [7]. Furthermore, the studies of Bajaj *et al*., confirmed that ZWC could be used as a new biocompatible pharmaceutical excipient and a functional biomaterial [8].

### 2.4. Mucoadhesiveness

Chitosan is a bioadhesive substance with vital applications due to its excellent mucoadhesive properties, when in a swollen state, based on its cationic character. The mucoadhesiveness of chitosan derives from non-covalent interactions between chitosan and mucin, such as electrostatic interactions and hydrogen bonds [48,49]. As a polycationic polymer with a high density of positive charges, it can adhere to both hard and soft tissues, such as epithelial and mucosal tissues, via hydration, hydrogen bonding and ionic interactions, and has been widely explored as drug carriers, especially for colon-specific delivery [50]. For instance, in the *in vitro* mucoadhesive tests, the prednisolone loaded alginate/chitosan microparticles prepared by the one-step method exhibited excellent mucoadhesiveness, whereas their other properties were not statistically significant different [51]. Recently, a new conception has been proposed for novel applications of chitosan. Fernandes *et al*., designed chitosan microspheres so as to serve as binders for *Helicobacter pylori* when facing a *H. pylori* gastric infection treatment [52], meaning that, after oral administration, the chitosan microspheres would remove *H. pylori* from infected patients, taking also the advantages of muco-bacterial adhesive properties.

### 2.5. Biodegradability

Chitosan is considered to be biodegradable in animal’s metabolism, as it can be degraded by enzymes which hydrolyze glycosidic bonds, like -GlcN-GlcN-, -GlcN-GlcNAc-, -GlcNAc-GlcN- and -GlcNAc-GlcNAc- linkages. It could also be hydrolyzed by certain human enzymes, especially lysozyme [53]. Besides, chitosan and its WSC-derivatives are promising biomaterials whose glycosidic bonds could be hydrolyzed in the human colon [3,54,55]. Based on the specific microflora of the colon-ecosystem, WSC-derivatives may be particularly hydrolyzed by β-glucosidase secreted by the colonic bacteria [56]. Additionally, it has already been confirmed that the biodegradability of chitosan in living organisms depends on the deacetylation degree and on its molecular weight [57,58,59].

## 3. Performances of PEC Based on Chitosan and WSC

PECs are polymeric materials chemically formed by polyelectrolytes of opposite charges. They can be fabricated with polycationic and polyanionic macromolecules or polyelectrolytes and surfactants with opposite charges [60]. Based on the favorable properties of chitosan/WSC, the PECs formed by chitosan/WSC and anionic substrates (such as sodium alginate, hyaluronic acid, pentasodium tripolyphosphate) may present many excellent performances, as following: (1) Good hydrophilic and swellable character; (2) Low interfacial tension and high permeability; (3) Excellent biodegradability; (4) Good biocompatibility; (5) Favorable film-forming behavior.

The good hydrophilic and swellable characters of PECs are due to their ability to swell in water and biological fluids, and retain a significant fraction of water within their latticed structures [61,62]. Their low interfacial tension and higher permeability make PECs well-suited biomaterials for the preparation of targeted/controlled drug release carriers. Due to an excellent biodegradability in the colon and good biocompatibility with organisms, PECs could be good candidates for designing new oral colon-specific drug delivery systems (OCDDS). More importantly, because of their favorable film-forming behavior, the drug carriers prepared with PECs might be the base for various formulations, such as films, capsules, microcapsules, microparticles or nanoparticles.

## 4. Drug Carriers Designed with PECs Based on Chitosan and WSC

### 4.1. Films

PECs, based on chitosan and WSC, might be used as the controlled release drug carriers for designing new skin drug delivery systems [63]. As can be seen in Table 2, some cross-linking agents, like glycerol and PEG200, were used to improve the performances of PEC films which were most of the times prepared via casting but also by self-assembly methods using some proper templates [63,64,65,66].

**Table 2:** Drug carriers prepared with films of polyelectrolyte complexes (PECs).

Polycationic Polyelectrolyte	Polyanionic Polyelectrolyte	Cross-Linking Agent	Preparation Method	Package Drugs	Reference
Chitosan	Polyacrylic acid	Glycerol/	Cast	—	[63]
		PEG200/			
		Hydrovance/			
		Trehalose			
Chitosan	NaCS	—	Cast	Paracetamol/5-ASA	[59,64]
Chitosan	Polyalkyleneoxide-maleic acid copolymer	—	Cast	Salicylic acid/Phenol	[65]
Chitosan	Hyaluronic acid	—	Self-assembly	—	[66]

**Figure 2:**
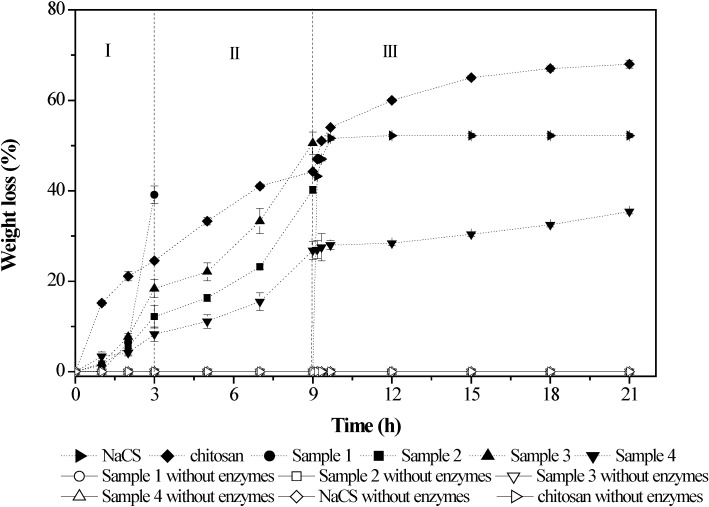
*In vitro* degradation profiles of chitosan/NaCS films in simulated gastric fluid (SGF, stage I), simulated intestinal fluid (SIF, stage II) and simulated colonic fluid (SCF, stage III). Sample 1: 563.3 kDa chitosan and 169.7 kDa NaCS; sample 2: 563.3 kDa chitosan and 31.2 kDa NaCS; sample 3: 135.3 kDa chitosan and 710.8 kDa NaCS; sample 4: 563.3 kDa chitosan and 710.8 kDa NaCS. Modified and cited from Zhu *et al.* [59].

**Figure 3:**
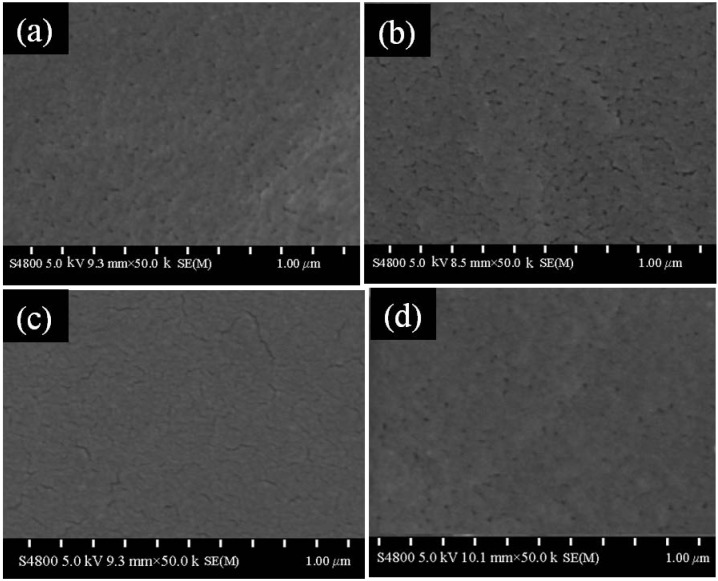
SEM morphology of chitosan/NaCS films prepared with different mass ratios. Mass ratios of chitosan to NaCS of (**a**) 1:4; (**b**) 1:2; (**c**) 3:4 and (**d**) 1:1. Chitosan with molecular weight 563.3 kDa and NaCS with molecular weight 710.8 kDa were used. Modified and cited from Zhu *et al.* [64].

In a previous study, Zhu *et al*., confirmed that the PEC films based on chitosan (molecular weight of 135.3 kDa) and NaCS (molecular weight of 710.8 kDa) showed the highest susceptibility to the hydrolysis by pepsin, amylase and trypsin. In addition, the disintegration time of the PEC films along the gastrointestinal tract (GIT) was different depending on the PEC formulations (Figure 2) [59]. The mass ratios of chitosan to NaCS had great influence on the morphology of the formulations and had important effects on the swelling properties and permeability of the films (Figure 3). A study on the release of paracetamol-loaded PEC films showed that the permeability of the films was closely related to the swelling properties and significantly influenced by the mass ratios, molecular weights and pH values [64]. These results indicated that the PEC films could be used as good candidates for the GIT delivery systems, especially for designing new colon-specific drug delivery systems.

### 4.2. Hard Hollow Capsules

Hard hollow capsules made up of chitosan have been a research concern since the 1990s [55]. Due to the solubility of chitosan in acidic conditions, hard hollow capsules prepared with chitosan must be coated with enteric coatings, like hydroxypropyl methylcellulose phthalate (HPMCP), to prevent disintegration during their passage through the stomach and small intestine [67]. These kinds of chitosan capsules proved to be useful carriers for colon-specific delivery of drugs like *n*-dodecyl-β-d-maltopyranoside and rebamipide, and could increase the effects of drugs by enhancing their absorption by the intestinal membranes [68,69].

**Figure 4:**
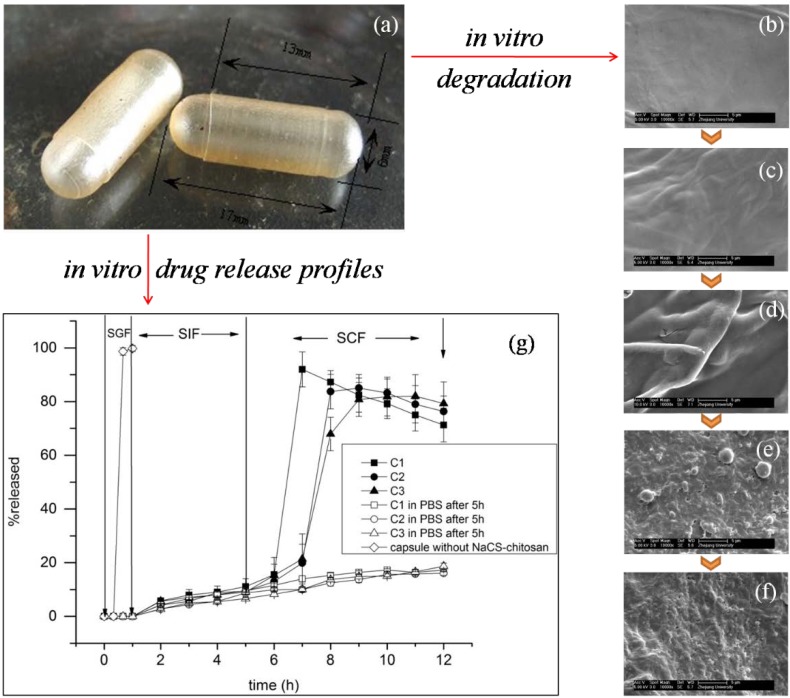
SEM morphology of the capsules and films, and the *in vitro* drug release profiles based on NaCS-chitosan. (**a**) Hard hollow capsules; (**b**) Films at 0 h in the *in vitro* experiment; (**c**) Films at 1 h in the *in vitro* experiment (SGF); (**d**) Films at 5 h in the *in vitro* experiment (SIF); (**e**) Films at 11 h in the *in vitro* experiment (SCF); (**f**) Films at 17 h in the *in vitro* experiment (SCF); (**g**) *In vitro* drug release profiles of 5-ASA from drug-loaded capsules based on NaCS-chitosan films or not. Stage I, in the SGF for 1 h; stage II, in the SIF for 4 h; stage III, in the SCF for 7 h. Sample C1(■), sample C2(●), sample C3 (▲), sample C1 in PBS after 5 h (□), sample C2 in PBS after 5 h (○), sample C3 in PBS after 5 h (Δ), capsule without NaCS-chitosan (◊). Samples (C1, C2, C3) were prepared with NaCS-chitosan films, gelatin and carrageenan as raw materials; capsules without NaCS-chitosan were prepared with gelatin and carrageenan as raw materials by the same method. Modified and cited from Wang *et al.* [18].

However, hard hollow capsules prepared with PEC and based on chitosan were seldom reported. In the past few years, Wang *et al*., developed a novel PEC capsule system which was formed by chitosan and sodium cellulose sulfate (NaCS) [18]. The PEC-based hard hollow capsules had a relatively homogeneous and smooth morphology (Figure 4a). *In vitro* degradation studies showed that the PEC films could be degraded by colon microflora and hydrolyzed in simulated gastrointestinal fluids, like simulated gastric (SGF) and intestinal fluids (SIF) (Figure 4b–f). More importantly, this kind of PEC-based hard hollow capsules loaded with 5-ASA may release about 80% of the drug in the simulated colonic fluid (SCF) during 4 h, indicating an excellent microflora-activated and colon-specific performance (Figure 4g). All these results indicated that the PEC-based capsules could be good candidates for designing new colon-specific drug delivery systems [18,70].

### 4.3. Microcapsules

Microcapsules of PEC based on chitosan and WSC, have gained large attention due to their potential applications (Table 3). With a large surface area, PEC microcapsules were designed in order to carry various drugs for targeted/controlled release [71,72,73,74,75,76]. Besides, they have also been widely studied in several biotechnological fields, such as fermentation with immobilized cells [77] and as vehicles for delivering probiotic bacteria [78,79].

**Table 3:** Drug carriers prepared with polyelectrolyte complexes (PEC) microcapsules.

Polycationic Polyelectrolyte	Polyanionic Polyelectrolyte	Cross-Linking Agent	Preparation Method	Package Drugs	Reference
Chitosan	NaCS	Sodium polyphosphate	Orifice-polymerization	5-ASA	[71]
Chitosan	Sodium alginate	—	Electrospray	Albumin	[72]
Chitosan	Sodium alginate	Calcium chloride	Modified orifice	Albendazole	[73]
Chitosan	NaCS	—	Self-assembly	—	[74]
Chitosan	*kappa*-carrageenan	Glutaraldehyde/Genipin/Tannic acid	Emulsion	Neem Seed Oil	[75]
WSC^b^	NaCS	Sodium polyphosphate	Orifice-polymerization	Lactoferrin	[76]

**Figure 5:**
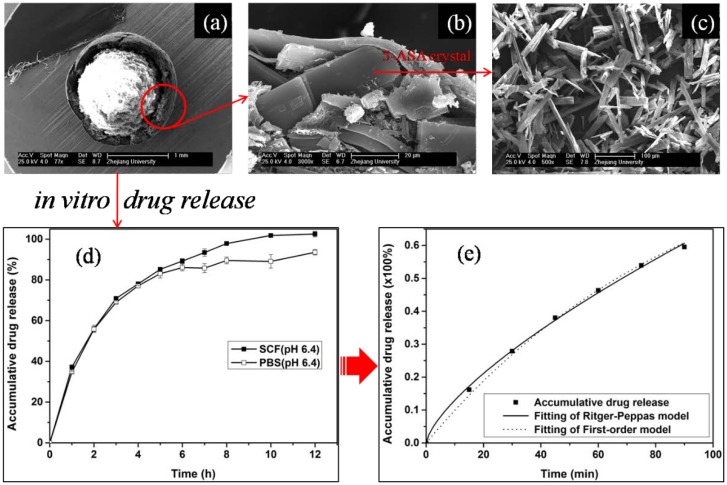
SEM morphology of the microcapsules and pure 5-ASA, and the *in vitro* drug release profiles. (**a**) Transection image of the double-walled capsule; (**b**) Transection image of the outer coated membrane layer (outer wall) and the loaded drug of 5-ASA; (**c**) Pure 5-ASA; (**d**) *In vitro* drug release studies in SCF (pH 6.4) and PBS (pH 6.4); (**e**) Nonlinear curve fitting of accumulative drug release in SCF (pH 6.4). Microcapsules were prepared with NaCS, WSC^b^ and PPS as raw materials. Modified and cited from Wu *et al.* [71].

Recently, novel PEC microcapsules based on NaCS, chitosan/WSC and sodium polyphosphate (PPS) were developed following simple processes, and this type of microcapsules could be used for designing new colon-specific drug delivery systems [24,71,76]. In this system, NaCS is the polyanion with –SO_3_^−^ groups while chitosan/WSC is the polycation with –NH_3_^+^ groups, PPS was used as a cross-linking agent. Based on the PEC obtained by ionization, spherical microcapsules were prepared by the orifice-polymerization method. These materials had been successfully used to encapsulate two kinds of model drugs: 5-aminosalicylic acid (5-ASA, a small molecular drug) [71] and lactoferrin (LF, a protein drug) [24,76].

It was verified that the PEC microcapsules loaded with 5-ASA had a relatively high loading efficiency (60.77%) and encapsulation efficiency (90.03%). SEM micrographs showed that the microcapsules were involved by a double-walled structure (shell) (Figure 5a,b). SEM transection images showed that 5-ASA entrapped in the microcapsule was in a crystal form (Figure 5b,c). *In vitro* release analysis showed that the drug was completely released in the simulated colonic fluid (SCF, pH 6.4), and the drug release was under the mechanism of anomalous transport (Figure 5d,e) [71].

**Figure 6:**
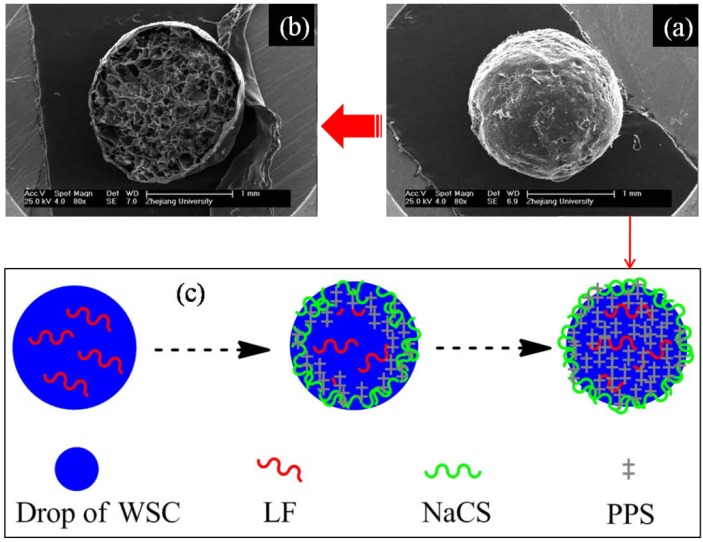
SEM morphology of the LF loaded NaCS-WSC-PPS microcapsules, and its formation process. (**a**) External appearance; (**b**) Transection image; (**c**) Schematic illustration of polyelectrolyte complexes formation process. Modified and cited from Wu *et al.* [24].

The microcapsules made of PEC and loaded with LF were also successfully prepared by Wu *et al.* [24,76]. SEM studies showed that the PEC microcapsules had a typical wall-capsule structure with smooth surfaces (Figure 6a,b). Fourier transform infrared spectroscopy (FT-IR) spectra analysis indicated that –NH_3_^+^ of chitosan/WSC, –SO_3_^−^ of NaCS and –[P_2_O_5_^4−^]– of PPS may react to form PEC. A schematic illustration of polyelectrolyte complexes formation process can be seen in Figure 6c. Drug loading and encapsulation efficiency studies also showed that the PEC microcapsules had a relatively high loading efficiency (49.06%) and encapsulation efficiency (86.3%). *In vitro* release studies showed that the microcapsules had a regular drug release behavior and the drug was released sustainably and completely in SCF (pH 5.5–7.0) [24].

**Table 4:** Drug carriers prepared with microparticles or nanoparticles of polyelectrolyte complexes (PEC).

Polycationic Polyelectrolyte	Polyanionic Polyelectrolyte	Cross-Linking Agent	Preparation Method	Package Drugs	Reference
Chitosan	Dextran sulfate	—	Self-assembly	Insulin	[80]
WSC^c^	Sodium alginate	Calcium chloride	Coaxial air-flow	Naproxen	[10]
Chitosan	Pectin	Tripolyphosphate	Emulsion	Gliclazide	[81]
Chitosan	Hyaluronan sodium salt	—	Stirring (Non-solvent)	Chloramphenicol succinate sodium salt/Cefotaxime sodium salt	[82]
Chitosan	Polybetaine	—	Stirring	Chloramphenicol succinate sodium salt	[83]
Chitosan	Hyaluronic acid	—	Self-assembly	Paclitaxel	[84]
Chitosan	Carboxymethyl gum kondagogu	—	Coacervation	Ofloxacin	[85]
Chitosan	Sodium alginate	—	Ionic gelation	Amoxicillin	[86]

WSC^c^: oligochitosan with deacetylation degree >85% and molecular weight around 3 kDa.

### 4.4. Microparticles/Nanoparticles

Microparticles or nanoparticles of PEC based on chitosan and WSC are also popular forms of drug carriers, as can be seen in Table 4. These are the most common strategies to improve the bioavailability of protein drugs via micro- and nanoencapsulation techniques. For example, in order to improve insulin bioavailability, Balabushevich *et al*., developed a kind of multifunctional protein-polymer microparticles via self-assembly method [80]. When using this multicomponent of insulin loaded microparticle system, the oral bioavailability of loaded-insulin improved due to the cumulative effect of each component, and the blood glucose level effectively lowered in the diabetic rats.

Besides, chitosan was usually used to coat hyaluronic acid-paclitaxel nanoparticles to prepare pH-responsive PEC drug-loaded-nanoparticles [84,87]. It is well-known that paclitaxel is a mitotic inhibitor used in cancer chemotherapy [88,89]. This kind of PEC nanoparticles takes several favorable advantages, such as a simple and feasible process, with a targeting and pH-sensitive release. For instance, the formulation of ofloxacin loaded nanoparticles provided a sustained drug release with 27% of the drug getting released in 12 h in the *in vitro* tests [90]. Guo *et al*., designed a kind of novel polyelectrolyte complex nanoparticles (PCNs) which are capable of associating bovine serum albumin (BSA), and *in vitro* studies showed that the system could keep a sustained drug release manner for 1 month without burst release [91]. Anitha *et al*., developed a combinatorial nanomedicine of 5-FU and curcumin (CUR) loaded nanoparticles, *in vitro* drug release profile showed a sustained release over a period of 4 days, further *in vivo* experiments in mouse verified an improved plasma concentration which could be prolonged up to 72 h [92]. Therefore, microparticles or nanoparticles of PEC based on chitosan and WSC could potentially be used in vital applications, such as designing of targeted/controlled drug delivery systems.

## 5. Conclusions

In view of the vital properties, such as no-toxicity, high biocompatibility and excellent biodegradability, chitosan and its water soluble derivatives (WSC) will be challenging biomaterials with potential applications in pharmaceutical fields, especially in the targeted/controlled release drug delivery field. Further, based on the favorable performances, such as good hydrophilic and swellable character, low interfacial tension and high permeability and favorable film-forming behavior, chitosan-based polyelectrolyte complexes, in proper forms like films, capsules and microparticles, might become important drug carriers with a promising application prospect.
